# Sacral Neuromodulation for Refractory Bladder Pain Syndrome/Interstitial Cystitis: a Global Systematic Review and Meta-analysis

**DOI:** 10.1038/s41598-017-11062-x

**Published:** 2017-09-08

**Authors:** Junpeng Wang, Yang Chen, Jiawei Chen, Guihao Zhang, Peng Wu

**Affiliations:** Department of Urology, Nanfang Hospital, Southern Medical University, Guangzhou, 510515 China

## Abstract

Bladder pain syndrome/interstitial cystitis (BPS/IC) is a common debilitating disease and there has not been consistently effective treatment. We aimed to evaluate all available literature regarding the efficacy and safety of sacral neuromodulation (SNM) for refractory BPS/IC. A comprehensive search of Pubmed, Web of Science and Cochrane Library through May 2016 was conducted. A total of 17 studies enrolling 583 patients were identified. Pooled analyses demonstrated that SNM was associated with great reduction in pelvic pain (weighted mean difference [WMD] −3.99; 95% confidence interval [CI] −5.22 to −2.76; *p* < 0.00001), Interstitial Cystitis Problem and Symptom Index scores (WMD −6.34; 95% CI −9.57 to −3.10; *p* = 0.0001; and WMD −7.17; 95% CI −9.90 to −4.45; *p* < 0.00001, respectively), daytime frequency (WMD −7.45; 95% CI −9.68 to −5.22; *p* < 0.00001), nocturia (WMD −3.01; 95% CI −3.56 to −2.45; *p* < 0.00001), voids per 24 hours (WMD −9.32; 95% CI −10.90 to −7.74; *p* < 0.00001) and urgency (WMD −1.08; 95% CI −1.79 to −0.37; *p* = 0.003) as well as significant improvement in average voided volume (WMD 95.16 ml; 95% CI 63.64 to 126.69; *p* < 0.0001). The pooled treatment success rate was 84% (95% CI 76% to 91%). SNM-related adverse events were minimal. Current evidence indicates that SNM might be effective and safe for treating refractory BPS/IC.

## Introduction

Bladder pain syndrome/interstitial cystitis (BPS/IC) is a chronic distressing disease, characterized by persistent or recurrent pelvic pain perceived to be related to bladder filling, associated with at least one other lower urinary tract symptom, without the evidence of a distinctively identifiable cause^1^. A recent report demonstrates BPS/IC is more common than previously thought, with a prevalence of 6.5% in adult females in the United States^2^. BPS/IC has a dramatic impact on patients’ quality of life^3, 4^. It often results in behavioral, emotional, psychological and even social problems^4^.

It has been reported that there are more than 180 treatments available for BPS/IC, but the results are usually varied^5^. It is estimated that 10% of patients with BPS/IC would progress to severe stage, refractory to conservative therapies^6^. The options to manage refractory BPS/IC include cystectomy with urinary diversion and bladder augmentation. Unfortunately, these interventions are associated with significant complications and often fail to alleviate the severity of pain^7^.

Sacral neuromodulation (SNM), introduced as a minimally invasive procedure in the 1980s, has previously been approved by the Food and Drug Administration to manage intractable overactive bladder symptoms and non-obstructive urinary retention^8, 9^. The transforaminal sacral afferent roots are stimulated by an implantable lead and electrode.SNM might also be a valuable option for patients with refractory BPS/IC. Several studies examining the effect of SNM in patients with refractory BPS/IC have been reported, but most included very few subjects and the results were conflicting^10–15^. Therefore, we systematically searched and analyzed published literature on the efficacy and safety of SNM in the treatment of refractory BPS/IC.

## Results

**Figure 1:**
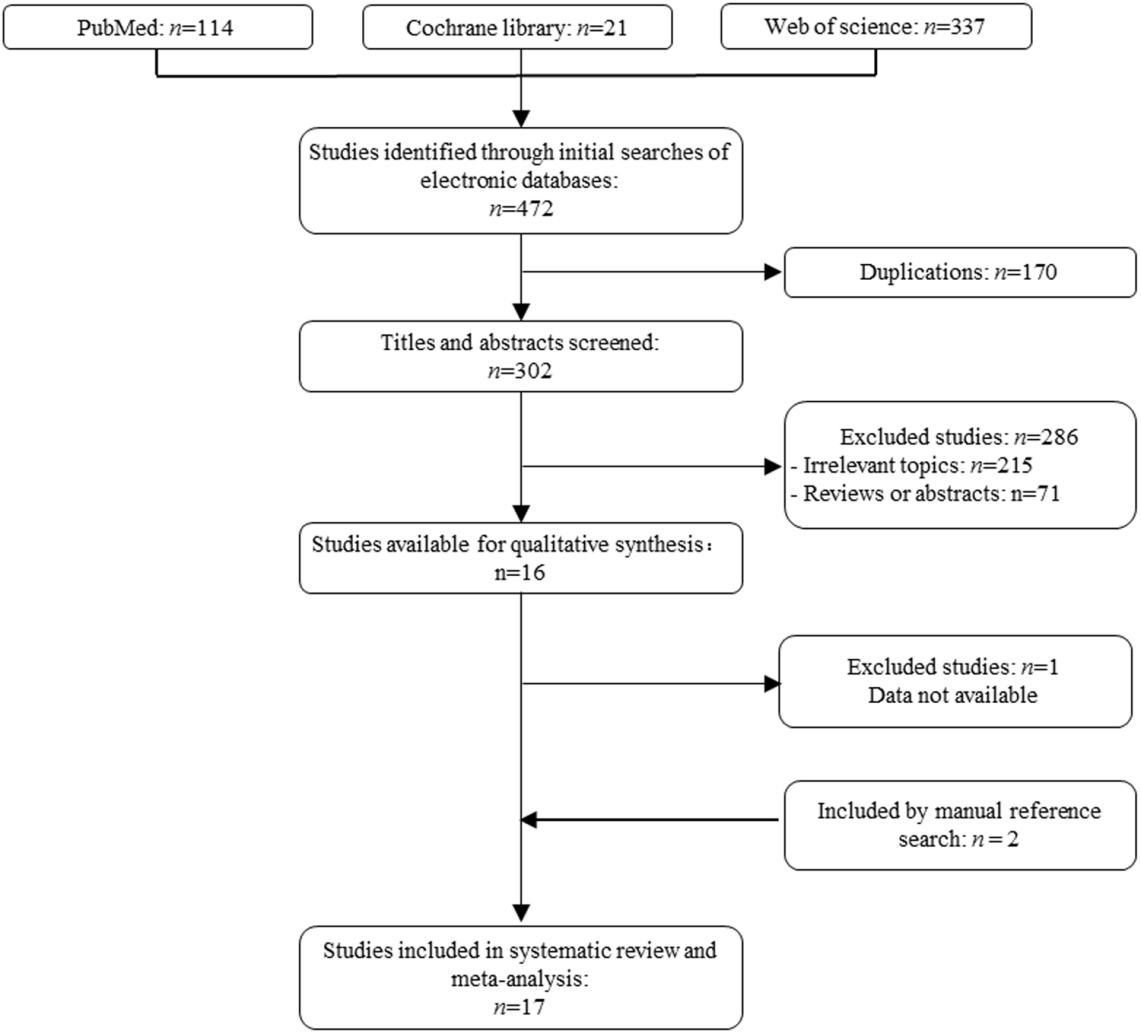
Flow diagram of studies included and excluded.

After screening 472 articles, 17 studies including 583 subjects were included in the final analysis (Fig. 1), one RCT^13^, eight prospective cohort studies^12, 14, 16–21^ and eight retrospective case series^10, 11, 15, 22–26^.

### Description of eligible studies

The main characteristics of the 17 studies are summarized in Table 1. BPS/IC was diagnosed using the National Institute of Diabetes and Digestive and Kidney Diseases (NIDDK) criteria^12, 14–16, 18, 24–26^, or by clinical criteria and cystoscopic findings^10, 11, 13, 17, 19, 21, 27^. Two studies did not state their diagnostic criteria^20, 23^. All patients included in the study had failed conservative management, with a BPS/IC duration ranging from 3 to 9.1 years. All studies aimed to assess the efficacy and safety of SNM in the treatment of refractory BPS/IC. The participants were predominantly female (89%, 519 of 583) and the follow-up time ranged from test stimulation to 86 months.

**Table 1:** Characteristics of eligible studies.

Study	Diagnostic criteria	Technique	Design	Patiens, no.	Mean age, yr	Mean duration of BPS/IC, yr	Women, %	Follow-up, mo
Chai 2001	NIDDK	Bilat PNE S3	P	6	NA	NA	83	—
Comiter 2003	NIDDK	Unilat staged S3	P	25	47	NA	96	14
Gajewski 2011	Clinical and cystoscopic	Unilat staged S3	R	78	42.37	4.1	90	61.5
Ghazwani 2011	NIDDK	Unilat staged S3	R	21	44.3	3	100	71.5
Kessler 2007	Clinical and cystoscopic	Unilat staged S3	P	209	NA	NA	87	24
Lavano 2006	Clinical and cystoscopic	Unilat staged S3/S4 + Bilat staged S3/S4	P	7	NA	NA	83	14
Maher 2001	NIDDK	Bilat PNE S3	P	15	62	5.2	100	—
Marinkovic 2011	Clinical and cystoscopic	Unilat staged S3	R	34	41	9.19	100	86
Peters 2003	NIDDK	Unilat staged S3	R	26	45	NA	81	5.6
Peters 2007	Clinical and cystoscopic	Pudendal vs unilat staged S3	RCT	22	44.8	5.9	86	6
Powell 2010	Clinical and cystoscopic	Unilat staged S3	R	39	54.4	NA	82.1	60
Siegel 2001	Clinical and cystoscopic	Unilat staged S3 + Unilat staged S4	P	10	44.5	4.6	90	29.5
Sokal 2015	NA	Staged S2, S3, and S4	R	9	50.5	8.5	100	12
Steinberg 2007	NIDDK	Bilat staged S3	R	15	43.2	3.8	NA	14.1
Whitmore 2003	NIDDK	Bilat PNE S3	P	33	44	4.1	100	—
Yang 2006	NIDDK	Unilat staged S3	R	4	52	NA	75	6
Zabihi 2008	NA	Bilat staged S2–S4	P	30	46.3	NA	70	15

PNE - percutaneous nerve evaluation; NIDDK - National Institute of Diabetes and Digestive and Kidney Diseases; NA - not applicable; RCT - randomized controlled trail; P - prospective; R - retrospective.

**Table 2:** Overall analysis of the outcome of SNM for refractory BPS/IC.

Outcomes of interest	Studies, no.	Baseline no. pts	Follow up no. pts	WMD (95% CI)	p value*	Study heterogeneity			
						*χ*2	df	*I* ^*2*^, %	p value*
Primary outcomes									
VAS score	12	188	185	−3.99 (−5.22 to −2.76)	**<0.00001**	135	11	92	**<0.00001**
ICPI	3	51	51	−6.34 (−9.57 to −3.10)	**0.0001**	8.48	2	76	**0.01**
ICSI	3	51	51	−7.17 (−9.90 to −4.45)	**<0.00001**	5.68	2	65	0.06
Success rate, %	10	258	258	84 (76 to 91)	—	—	9	56.7	**0.014**
Secondary outcomes									
Daytime frequency	4	58	58	−7.45 (−9.68 to −5.22)	**<0.00001**	5.07	3	41	0.17
Nocturia	6	96	96	−3.01 (−3.56 to −2.45)	**<0.00001**	7.95	5	37	0.16
Voids per 24 hours	6	95	95	−9.32 (−10.90 to −7.74)	**<0.00001**	3.26	5	0	0.66
Urgency	4	61	61	−1.08 (−1.79 to −0.37)	**0.003**	27.8	3	89	**<0.00001**
AVV, ml	6	100	100	95.16 (63.64 to 126.69)	**<0.00001**	23.1	5	78	**0.0003**
Complication rate, %	14	345	345	3 (0 to 11)	—	—	13	0	1
Explantation rate, %	10	258	258	8 (3 to 13)	—	—	9	80.2	**<0.0001**

VAS - visual analog scale; ICPI - Interstitial Cystitis Problem Index; ICSI - Interstitial Cystitis Symptom Index; AVV - Average voided volume; WMD - weighted mean difference; pts - patients.

*Statistically significant results are shown in bold.

### Primary outcomes

#### Pelvic pain

**Figure 2:**
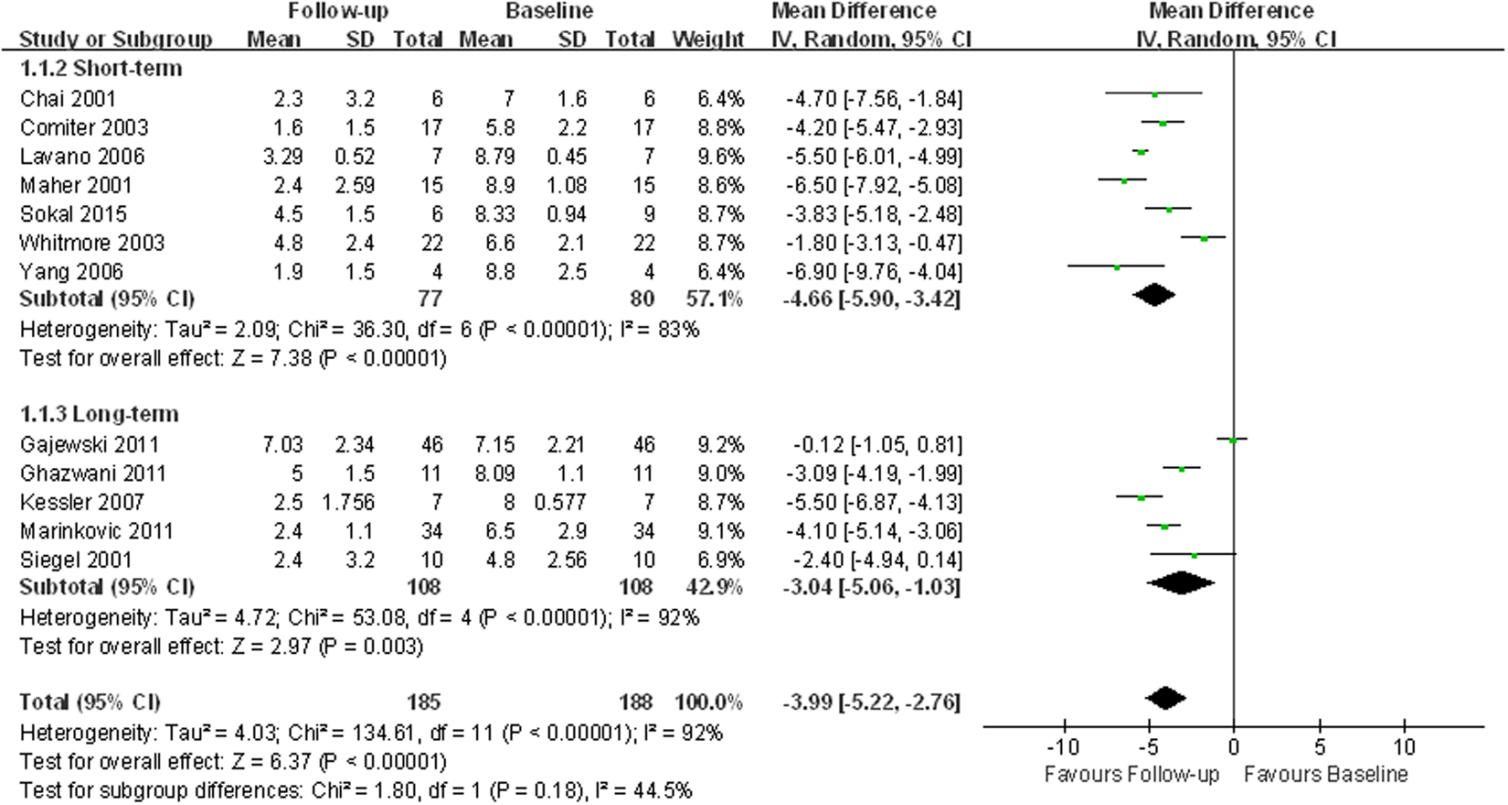
Forest plot of pelvic pain measured by visual analog scale score (SD = standard deviation; IV = inverse variance method; CI = confidence interval).

Twelve trials assessed pelvic pain using the VAS in 178 patients^10, 12, 14–19, 21–23, 26^. Pooled analysis showed that SNM significantly decreased VAS compared to baseline (WMD −3.99; 95% CI −5.22 to −2.76; *p* < 0.00001) (Fig. 2).

#### Interstitial cystitis problem index and interstitial cystitis symptom index

**Figure 3:**
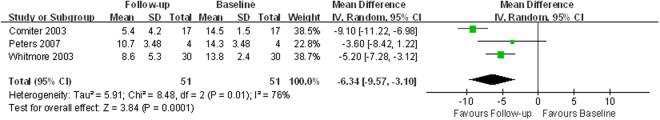
Forest plot of the Interstitial Cystitis Problem Index (SD = standard deviation; IV = inverse variance method; CI = confidence interval).

**Figure 4:**
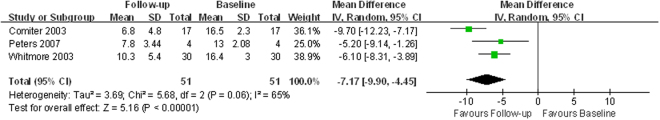
Forest plot of the Interstitial Cystitis Symptom Index (SD = standard deviation; IV = inverse variance method; CI = confidence interval).

One RCT and two prospective studies reported ICPI and ICSI scores^12–14^. Pooling the data of these three studies showed significant reduction in ICPI scores (WMD −6.34; 95% CI −9.57 to −3.10; *p* = 0.0001; Fig. 3) and ICSI scores (WMD −7.17; 95% CI −9.90 to −4.45; *p* < 0.00001; Fig. 4) following SNM.

#### Success rate

**Figure 5:**
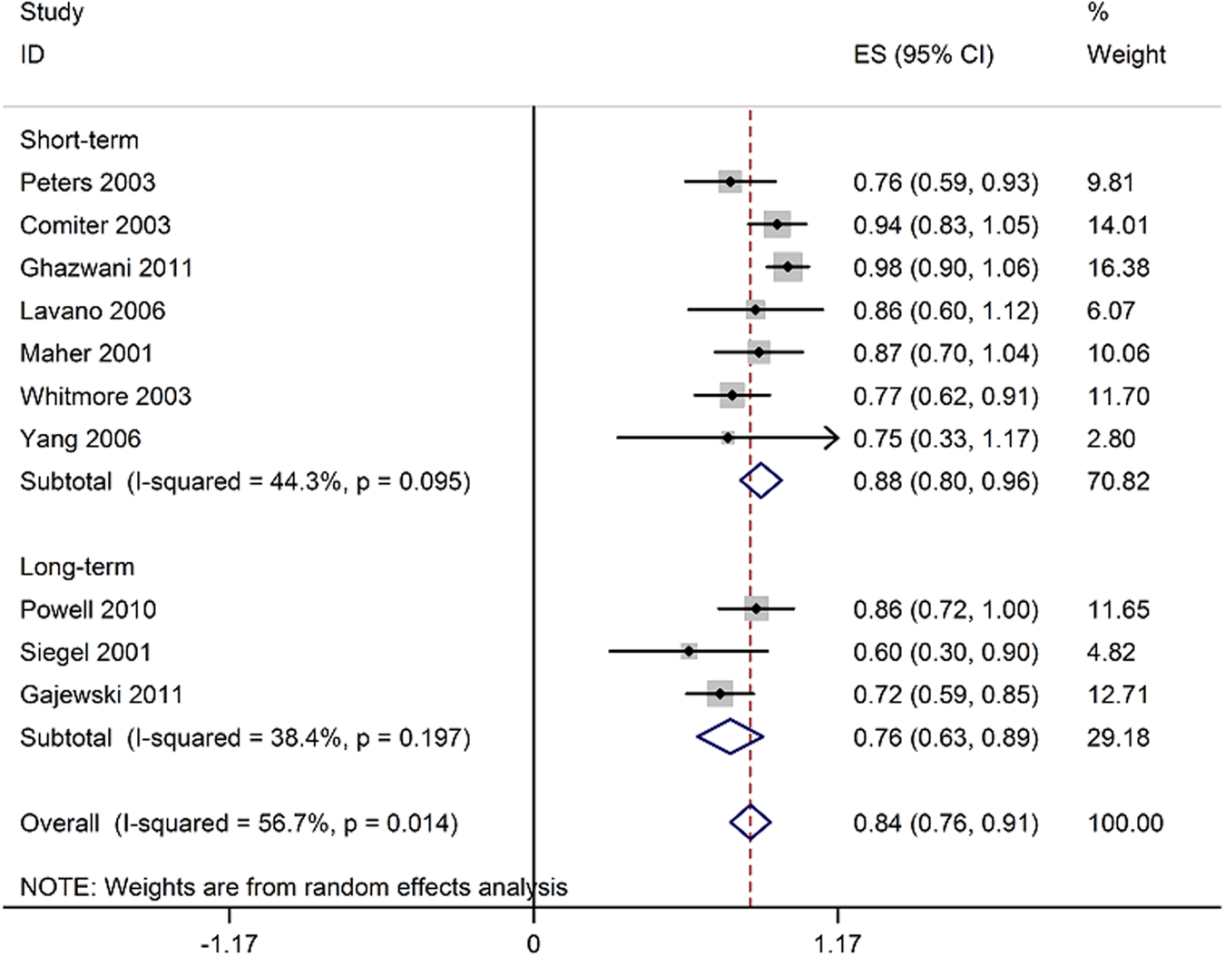
Forest plot of success rate (ES = effect size; CI = confidence interval).

Ten studies assessed the success rate in 258 patients that ranged between 60% and 98%^10–12, 14, 15, 18, 19, 21, 25, 26^, and pooled analysis demonstrated that the success rate was 84% (95% CI 76% to 91%; Fig. 5).

### Secondary outcomes

#### Daytime frequency, nocturia and voids per 24 hours

**Figure 6:**
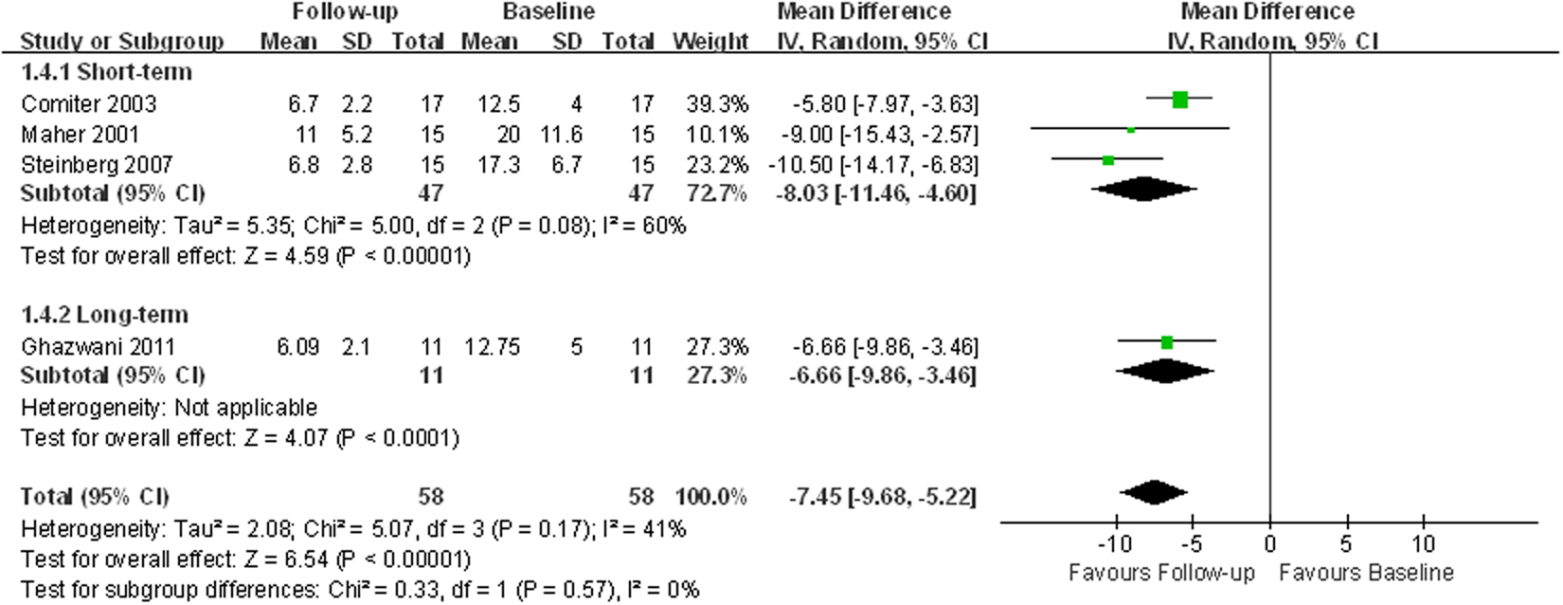
Forest plot of daytime frequency (SD = standard deviation; IV = inverse variance method; CI = confidence interval).

**Figure 7:**
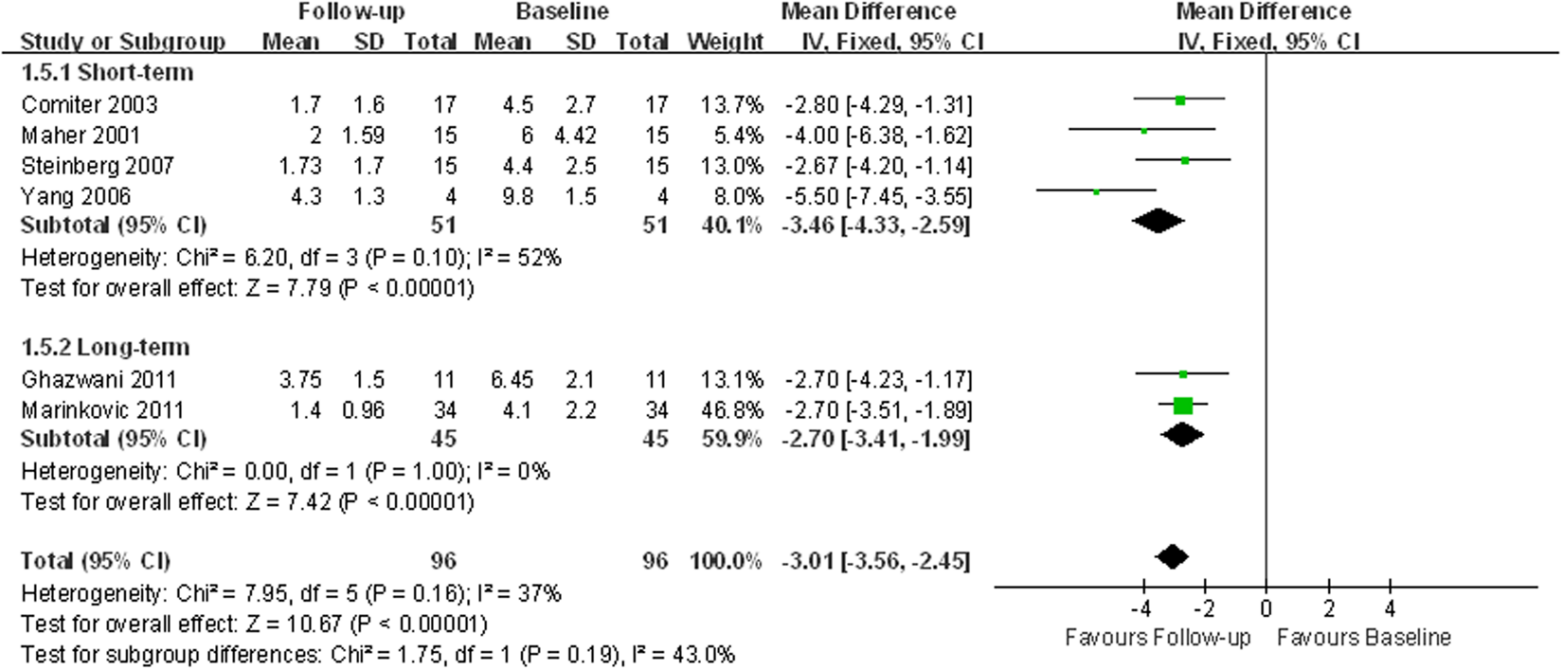
Forest plot of nocturia (SD = standard deviation; IV = inverse variance method; CI = confidence interval).

Four studies^12, 15, 18, 24^ assessed daytime frequencies and two other studies assessed nocturia^22, 26^. Pooled analysis detected significant improvement in both daytime frequency (WMD −7.45; 95% CI −9.68 to −5.22; *p* < 0.00001; Fig. 6) and nocturia (WMD −3.01; 95% CI −3.56 to −2.45; *p* < 0.00001; Fig. 7). Pooling the data of six studies that reported voids per 24 hours demonstrated a significant difference favoring SNM (WMD −9.32; 95% CI −10.90 to −7.74; *p* < 0.00001; Table 2, Supplemental Fig. S1), consistent with the results of daytime frequency and nocturia^12–14, 16, 22, 26^.

#### Urgency

Four studies assessing the efficacy of SNM, included analysis of urgency^14–16, 18^. Pooling the data from these four studies demonstrated a significant reduction in urgency (WMD −1.08; 95% CI −1.79 to −0.37; *p* = 0.003; Table 2, Supplemental Fig. S2) following SNM.

#### Average voided volume

Six studies reported baseline and post-treatment AVV^10, 12–14, 22, 26^. The pooled data showed a significant improvement in AVV (WMD 95.16 ml; 95% CI 63.64 to 126.69; *p* < 0.0001; Table 2, Supplemental Fig. S3), following SNM.

#### Complication rate and explantation rate

**Figure 8:**
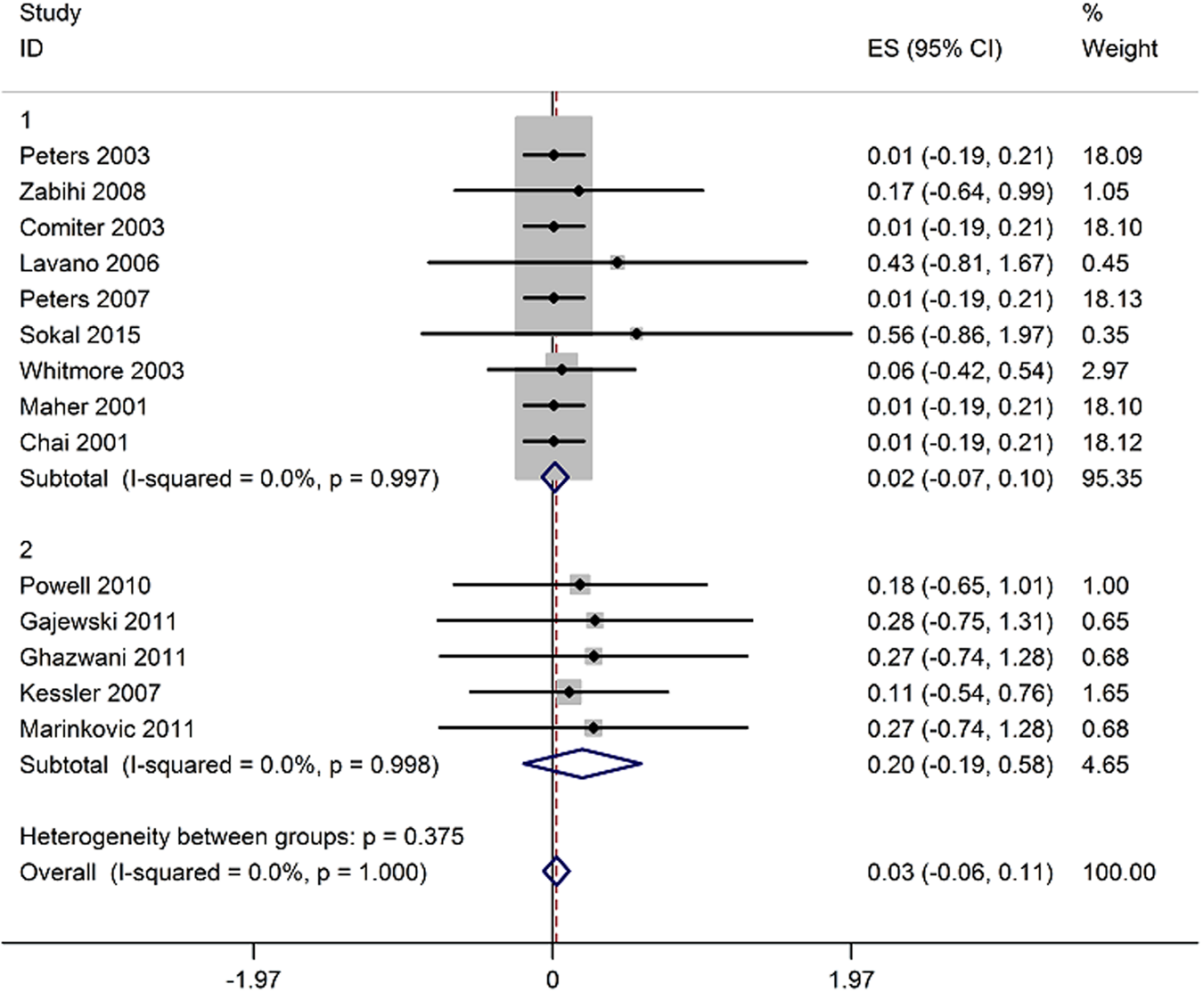
Forest plot of complication rate (ES = effect size; CI = confidence interval).

14 studies reported complication rates in 345 patients, ranging from 0% to 56%^10–18, 20–23, 25^. Pooling the data demonstrated an overall complication rate of 3% (95% CI 0 to 11%; Fig. 8). Pooling the data of 10 studies including 258 patients that reported explantation rate showed an overall explantation rate of 8% (95% CI 3% to 13%; Table 2, Supplemental Fig. S4)^10–13, 15, 17, 19–21, 25^.

#### Subgroup analysis

Short-term and long-term subgroup analyses were consistent with the original analysis. Short-term subgroup had a slightly higher success rate, slightly lower complication rate and explantation rate compared to the long-term subgroup.

#### Sensitivity analysis and meta-regression

Ten or more studies assessing SNM included analysis of VAS, success rate, complication rate and explantation rate. Sensitivity analyses did not show significant change in these results. In addition, meta-regression analyses did not detect significant correlations between publication year or type of study design and the above four outcomes.

#### Risk of bias and publication bias

The risk of bias was satisfactory in the included studies (Supplemental Fig. S5 and S6). The funnel plot for VAS was largely symmetric, demonstrating no obvious publication bias in this present meta-analysis (*p*-values from the Begg test and Egger test were 0.451 and 0.567, respectively) (Supplemental Fig. S7).

## Discussion

This meta-analysis of 17 studies assessing the efficacy of SNM in the treatment of BPS/IC demonstrated that SNM was associated with significant improvement in pelvic pain, ICPI and ICSI scores, as well as neutralizing other symptoms including daytime frequency, nocturia, urgency and average voided volume.

During the last few decades, various modalities have been introduced for treating BPS/IC, such as oral drug therapy, intravesical instillations and surgery, but they have not provided consistent clinical improvement or relief ^28, 29^.

Since the pathogenesis of BPS/IC is still unclear, current treatment protocols are aimed at alleviating the symptoms. Our meta-analysis suggests that SNM effectively alleviates pelvic pain, with an average decrease of 3.99 in VAS score. This finding is encouraging for patients, as pelvic pain seriously affects their quality of life and could lead to other symptoms such as daytime frequency and nocturia^1, 28^.

The ICPI and ICSI scores have been widely accepted as reliable and validated measures of BPS/IC symptoms^30^. Significant improvements in ICSI and ICPI scores demonstrate that SNM has significant influence on the subjective symptoms.

The pooled data demonstrated that SNM was associated with a significant decrease in daytime frequency, nocturia and voids per 24h. This is very important, as increased voiding frequency can lower productivity, lead to considerable anxiety and prevent participation in social activities.

SNM technique was introduced in the 1980s and has recently become a widely used therapy for refractory urgency-incontinence, overactive bladder symptoms and non-obstructive urinary retention^8, 9, 31^. Following failed conservative treatment, SNM is considered to be the therapeutic option of choice for lower urinary tract dysfunction, before resorting to more invasive surgeries such as bladder augmentation and urinary diversion.

The exact etiology of BPS/IC remains to be clearly identified. There is emerging evidence of neural abnormalities both peripheral and central in BPS/IC, which plays an important role in pain sensitivity, urgency, and frequency symptoms^27, 32^. Dysregulated nervous system may not only maintain the perception of pain following acute injury, but also magnify pain perception in response to stimulus^33^. Specifically the precise mechanism of symptom relief caused by SNM is still not fully understood and it is believed that SNM may inhibit the transmission of abnormal sensory signals to the spinal cord and brain by acting on the afferent pathways^34, 35^. Spinal cord stimulation has been used to manage chronic pain conditions, including chronic back pain, idiopathic angina pectoris and migraine, with success^34^.

From this meta-analysis we cannot conclude whether bilateral or unilateral SNM should be the preferred therapeutic option. Considering that only a few studies assessed bilateral SNM and enrolled small number of patients, we did not perform formal subgroup comparison of bilateral versus unilateral stimulation. Further well-designed RCTs are warranted to determine this issue.

It is probable that the long-term efficacy of SNM in the treatment of BPS/IC decreases significantly. However, the subgroup analysis did not show significant differences between short-term and long-term improvement in symptoms. Clearly defined therapeutic success is very important in evaluating SNM efficacy. Most studies utilized more than 50% improvement in pelvic pain/ voiding symptoms as the therapeutic goal. It has been reported that SNM may lose efficacy overtime^20^. However, in this present meta-analysis, long-term success rate was found to be 76%, similar to short-term success rate at 88%, indicating SNM could provide good long-term efficacy.

The safety of any treatment is always of great importance and pooled analysis indicates that SNM is safe for BPS/IC. Complications were minimal, and most of them were pain at the site of the implantable pulse generator (IPG), infection and lead migration or dysfunction, which usually can be treated effectively. However, there was a slight increase in long-term complications and this could be attributed to IPG malfunction due to accidents (e.g., trauma to the pelvic region). The overall explantation rate was relatively low and the most common reasons for removal were loss of efficacy, painful stimulation, depleted batteries and technical malfunction. It seems reasonable to speculate that explantation rate will decrease continually as technology improves.

Although the findings of this first meta-analysis aiming to access SNM as a therapeutic option in refractory BPS/IC are promising, the following limitations must be taken into account. The main limitation is that the sample sizes of all included studies were small. Eight of the studies were retrospective case series. In general, case series studies are prone to increased risk of selection bias. However, outcomes reported by these case series were similar to the RCT^13^. Moreover, complication rate and explantation rate were 0 in several studies and had to be imputed. To calculate the overall complication rate and explantation rate, we assumed it to be 0.01. Therefore, one should be cautious when interpreting the summary rate reported. However, because sensitivity analyses did not show that these studies had significant influence on the results, we consider this approach to be acceptable. Finally, there was substantial heterogeneity between studies as well as scarcity of data that made subgroup analyses of bilateral versus unilateral stimulation and stimulation parameters impossible. To the best of our knowledge, the present study is the first meta-analysis to assess the efficacy and safety of SNM as a therapeutic option for BPS/IC. We applied multiple rigorous search strategies, strictly evaluating criteria and subgroup analysis, sensitivity and meta-regression analysis. Hence, this meta-analysis provides strong evidence in this area.

## Conclusion

This meta-analysis indicates that SNM may be effective and safe for treating BPS/IC refractory to conventional therapies. This present study demonstrates marked improvements in not only pelvic pain, but also in voiding symptoms and improved symptom scores. Adverse events were minimal, transient and usually could be treated effectively and there were no irreversible or life threatening complications. In addition, the technique itself is completely reversible. However, considering the overall low quality of included studies, further well-designed, large-volume RCTs are required to reach definitive conclusions.

## Methods

This systematic review and meta-analysis was performed following Preferred Reporting Items for Systematic reviews and Meta-Analysis guidelines^36^. A pre-specified protocol including objectives, literature-search strategies, eligibility criteria, outcome measurements, quality assessment and methods of statistical analyses was prepared.

### Literature-search strategy

A systematic literature search was performed in May 2016 without any restrictions for languages, regions, or publication types. We searched the electronic databases of Pubmed, Web of Science and Cochrane Library, using the search terms sacral neuromodulation, electric stimulation therapy, sacral nerve stimulation, neuromodulation, SNM and associated those with the search terms painful bladder syndrome, bladder painful syndrome, interstitial cystitis and chronic pelvic pain. The reference lists of all identified studies and reviews were screened to select relevant articles.

### Inclusion and exclusion criteria

All available randomized controlled trials (RCTs), comparative studies and single-arm cohort studies that assessed effectiveness of SNM for treating BPS/IC, were considered to be eligible. Non-original articles, duplicate reports, case reports and studies using animal models were excluded.

### Data extraction and outcomes of interest

Two authors (J. P. Wang and Y. Chen) independently extracted and summarized the data from the selected articles, including study characteristics (diagnostic criteria, study type, and follow-up), number of participants, SNM (percutaneous nerve evaluation or a two-staged procedure, unilateral or bilateral) and outcomes (primary and secondary outcomes). Any discrepancies in the extracted data were resolved by the senior author (P. Wu).

The primary outcomes evaluated were the 0–10 Visual Analog Scale (VAS), the Interstitial Cystitis Problem Index (ICPI), the Interstitial Cystitis Symptom Index (ICSI) and success rate. The secondary endpoints included daytime frequency, nocturia, voids per 24 hours, urgency, average voided volume, complication rate and explantation rate.

### Assessment of risk of bias and statistical analysis

Risk of bias in RCTs was assessed using the Cochrane risk of bias assessment tool^37^. Methodological quality of comparative non-RCTs was assessed utilizing the described Cochrane tool and a predefined 8-point quality control measure^38^.

The meta-analyses were conducted using Review Manager 5.3 (Cochrane Collaboration, Oxford, UK) and the metan command of Stata v.12 (StataCorp, College Station, TX, USA). Weighted mean difference (WMD) was used for continuous outcomes and 95% confidence intervals (CIs) were applied for all outcomes. For studies that presented data as medians and range values, the means and standard deviations were calculated using the pragmatic approach described by Hozo *et al*.^39^.

Heterogeneity among studies was assessed by the chi-square test and I^2^ statistic. A *p* value < 0.10 or an I^2^ > 50% denoted the existence of significant heterogeneity. The fixed-effects model was used if no substantial heterogeneity was observed, otherwise, the random-effects model was used.

Subgroup analysis was performed to compare short-term and long-term effectiveness of SNM. Sensitivity analysis and meta-regression were performed to screen for potential sources of heterogeneity. Publication bias was assessed using funnel plots.

## Electronic supplementary material

Supplemental Figures
