# Supplementary material for: Sacral Neuromodulation for Refractory Bladder Pain Syndrome/Interstitial Cystitis: a Global Systematic Review and Meta-analysis
Source: Sci Rep. 2017 Sep 8;7:11031. doi: 10.1038/s41598-017-11062-x (PMC5591255; doi:10.1038/s41598-017-11062-x)
Supplement: Supplementary file 1 — Supplemental Figures [file 41598_2017_11062_MOESM1_ESM.doc]

**Sacral Neuromodulation for Refractory Bladder Pain Syndrome/Interstitial Cystitis: a Global Systematic Review and Meta-analysis**

**Junpeng Wang, Yang Chen, Jiawei Chen, Guihao Zhang, Peng Wu**

**Institute:** Department of Urology, Nanfang Hospital, Southern Medical University, Guangzhou, China

Supplementary Figure S1. Forest plot of voids per 24h.

Supplementary Figure S2. Forest plot of urgency.

Supplementary Figure S3. Forest plot of average voided volume.

Supplementary Figure S4. Forest plot of explantation rate.


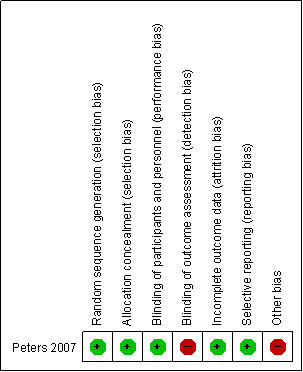


Supplemental Figure S5. Risk of bias of the included RCT.


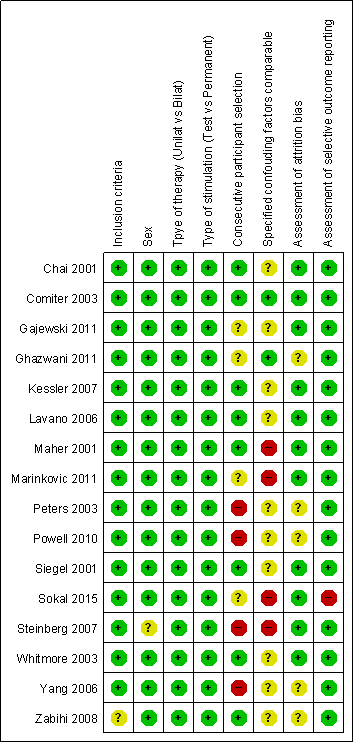


Supplemental Figure S6. Risk of bias of included non-RCTs.


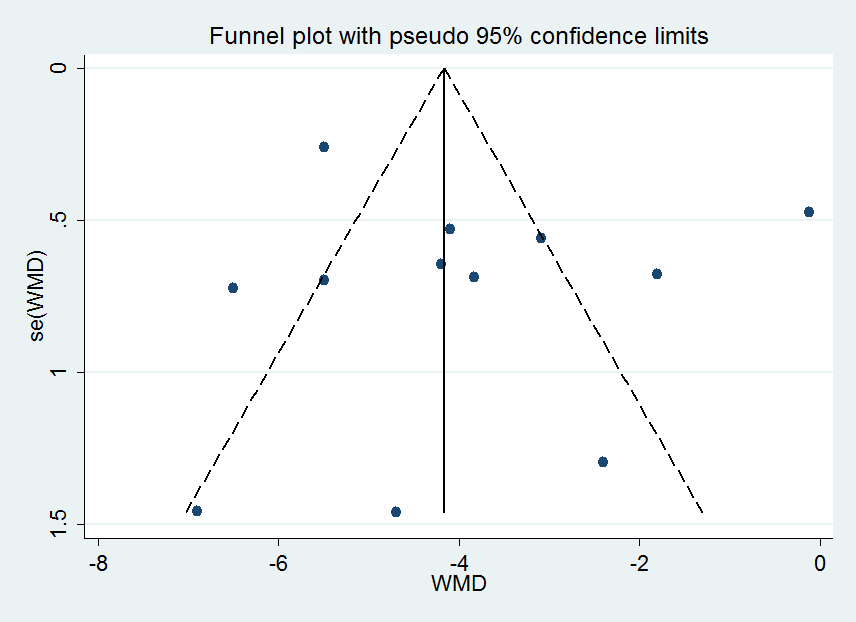


Supplemental Figure S7. Funnel plots of pelvic pain measured by visual analog scale score.
